# The importance of the concepts of disaster, catastrophe, violence, trauma and barbarism in defining posttraumatic stress disorder in clinical practice

**DOI:** 10.1186/1471-244X-8-68

**Published:** 2008-08-12

**Authors:** Luciana L Braga, Jose P Fiks, Jair J Mari, Marcelo F Mello

**Affiliations:** 1 Graduate Program in Psychiatry – Department of Psychiatry, Escola Paulista de Medicina – Universidade Federal de São Paulo (Unifesp), São Paulo, Brazil; 2 Department of Psychiatry, Escola Paulista de Medicina – Universidade Federal de São Paulo (Unifesp), São Paulo, Brazil

## Abstract

**Background:**

Several terms in the scientific literature about posttraumatic stress disorder are used with different meanings in studies conducted by different authors. Words such as *trauma, violence, catastrophe, disaster *and *barbarism *are often used vaguely or confusingly, and their meanings change in different articles. The lack of conceptual references for these expressions complicates the organization of literature. Furthermore, the absence of clear concepts may be an obstacle to clinical treatment because the use of these words by the patients does not necessarily point to a diagnosis of posttraumatic stress disorder.

**Discussion:**

A critical review of scientific literature showed that stress can be divided in stages to facilitate specific terminological adjustments to the event itself, to the subject-event interaction and to psychological responses. Moreover, it demonstrated that the varying concept of trauma expands into fundamental psychotherapeutic definitions and that the meanings of violence associated with barbarism are an obstacle to resilience. Therefore, this study updates the etymological origins and applications of these words, connects them to the expansions of meanings that can be operated in the clinical care of patients with posttraumatic stress disorder, and analyzes them critically according to the criterion A of DSM-IV and ICD-10.

**Summary:**

The terminology in the literature about posttraumatic stress disorder includes a plethora of terms whose meanings are not fully understood, and that, therefore, limit this terminology. The analysis of these terms suggested that the transformation of the concept of *trauma *led to a broader understanding of this phenomenon in its psychic dimensions, that a barbarian type of violence constitutes an obstacle to resilience, and that the criterion A of the DSM-IV and ICD-10 shows imprecision and conceptual fragilities.

**Methods:**

To develop this debate article, a current specialized literature review was achieved by searching and retrieving the key terms from two major databases: PubMed and PsycINFO. The key terms included "disaster", "catastrophe", "barbarism", "terrorism", "trauma", "psychic trauma" and "violence", also in combination with the terms "PTSD", "concept" and "conceptual aspects". The data were captured specially from review articles. The included studies were those mostly identified by the authors as relevant by the presence of a *conceptual approach *in any part of the paper. Researches that relied solely on empirical indicators, like psychopathological, neurobiological or pharmacological aspects, were excluded. The focus here was in conceptual aspects, even when some few empirical studies were included.

As it was noted a paucity of medical references related to conceptual aspects of these terms, a wider literature needed to be included, including chapters, books and articles proceeded from the Humanities areas. "Interdisciplinary research is needed in this area to include perspectives from a range of different disciplines" once that "to promote public health (...) new dimensions of such interactions and the implications thereof should be pursued in collaboration with researchers from broader areas" [1].

## Background

Posttraumatic stress disorder (PTSD) was defined as a psychiatric disease in 1980 and included in the third edition of the DSM by the American Psychiatric Association (APA) [2]. Considerable advances have been made in the research to understand PTSD physiopathologic and psychopathological mechanisms and to develop possible treatments. At a time when contradictions and questions arise in PTSD research [3-6], researchers in the area acknowledge the possible conceptual frailty of this diagnosis and search for a more consistent theoretical classification.

The plethora of terms used in PTSD literature, such as aggression, violence, disaster, catastrophe, barbarism, stressful event and trauma, do not point to a corresponding understanding of the meanings, applications and limits of these constructs. These terms are often vaguely used, meanings change in different studies, and there is a lack of conceptual references to guide their use in scientific literature. Some of the most common expressions define events according to consequences, such as in the case of the word "stressor", and not according to the characteristics of that single event [7,8].

An initial attempt to undo such terminological confusion is to refer to etymology and to retrace the path of the concept into clinical practice. Rather than to establish definitive positions about this topic, the purpose of this study is to promote a discussion of conceptual questions about PTSD terms. Therefore, this study presents definitions of catastrophe, disaster, trauma, violence and barbarism from a clinical perspective to clarify their scope and limitations.

## Discussion

### About the event

The word disaster has its origin in the Italian word *disastro *(dis + astro, "bad star") [9], and refers to an event marked by destruction, death, physical injury and human suffering that causes permanent changes to human societies, ecosystems and the environment [7]. "Disasters generate an array of individually and collectively experienced stressors of varying degrees of intensity that interact with multiple characteristics of the person and environment to produce diverse outcomes that evolve over time" [10].

Disasters tend to expose unselected populations to trauma, randomly. Within a given community, individuals can be directly, indirectly or remotely exposed to the event. The medical model focuses on specific intervention for each of these groups, aiming to prevention, healing and recovery of PTSD and other psychiatric disorders. On the other hand, the wellness models comprehend disasters from the distress challenge, focusing on restoring homeostasis [11]. Therefore, should disasters survivors be viewed as "psychologically damaged by the experiences that befell them or was it more appropriate to validate the experience of trauma from a humanistic and existential perspective by viewing their responses as an adaptation to frightening environmental events?" [12]. These points of view on disaster need not to be in conflict. Taken together, these models constitute a broader perspective, addressing the negative (distress symptoms, disease) and positive effects (resilience and post-traumatic growth) of disaster [11].

Ecological disasters may also be called catastrophes, events of great proportions usually associated with natural phenomena that cause death and destruction. The origin of *catastrophe *is Greek (*kata + strophein*) and its literal meaning was "overturn". According to its definition, it is an event that causes trauma [13] due to its capacity to destroy most of a community. Catastrophes are extreme events that cause PTSD in a large number of victims in the affected community, and are easily identified as events that cause physical suffering. Individual traumatic events, however, are only accessed by the healthcare professionals through the patient's narrative. Personal history, trauma impact on psyche, educational level, and other factors that compose the patient's subjective life determine the choice and use of different terms to describe the situation experienced.

A careful examination of the words used by patients is fundamental to understand the psychological impact of events that may trigger traumatic responses. Situations described as "catastrophic" may not necessarily indicate that the situation that the patients experienced was a "catastrophe", although it may have an equally devastating psychological representation.

The event alone has importance to the mental healthcare professional only when it is a situation that can produce psychopathological responses from the patient. The idea of a "disaster taxonomy" is based on the principle that there are variable emotional responses that depend on the type of disaster, the degree of personal impact, the size of the group affected, and the geographical and temporal range of the event [7].

The "disaster taxonomy" stresses the importance of avoiding overlapping or confusing terms to define events because their different aspects may trigger different psychological responses. The use of an adequate terminology requires the understanding of how a traumatic situation causes and becomes part of a stressful process, which may be divided intro three major stages: 1) environmental input in the form of an event; 2) immediate apprehension of the event; and 3) psychological responses after the event [8].

The first stage is restricted to the event itself, and may be objectively measured in number of victims, degrees in the Richter scale, or square kilometers of affected area. "Pure" words, without qualifiers, such as "event", "stimulus", "loss", "disaster" or "catastrophe" are used for such description. The second stage goes beyond the isolated event and incorporates the initial perceptions of the victim. Words such as "danger", "shock", "risk of death", "threat" or "stressor" illustrate the interaction between the stimulus and the person that experiences it. The third stage corresponds to the psychological response to the event, and words such as "mourning", "response to stress" or "trauma" are used [8]. Intense psychological responses are not always associated with concrete situations; for example, the imminence of a possible terror attack may produce an important effect on psyche and trigger a typical PTSD response.

### Trauma

The word *trauma*, originally used in medicine, has an Indo-European root with a double meaning: a) to rub, grind, perforate; and b) overcome, to go through [13,14]. *Trauma *is a violent shock that is capable of producing an impact that the individual cannot resist. Therefore, *trauma *that "perforates" is the same that makes "go through", which describe the two possible psychic developments seen in a traumatic situation: the development of PTSD or of resilience – the ability to go through trauma and to introject meaning into one's own life.

The formal recognition of PTSD in 1980 changed the conceptual understanding of *trauma*. In the 19^th ^Century, except in the psychoanalytic literature, the word *trauma *referred primarily to wounds or violent tissue rupture and had no psychological connotations. The hypothesis that a terrible event might cause effects other than those merely physical was developed in the 1860's, with the description of the "Railroad Spinal Syndrome" by John Eric Erichsen [15]. Since then, a proliferation of descriptive terminologies related to traumatic experiences emerged, many of which pointed to railway accidents or combat experiences, like "spinal concussion", "soldier's heart", "traumatic shock", "shell shock", "battle fatigue", "war psychoneurosis" etc. [16]

In 1882, Jean-Martin Charcot studied patients whose psychic symptoms appeared after severe trauma, such as train crashes or wars, and served as traumatic triggers in individuals that had a certain inherited predisposition or "*diáthese*". Therefore, he described the "*névrose traumatique" *or *"hystérie traumatique" *to classify these cases. Charcot concluded that a physical trauma could produce emotional disorders [17].

In *On the psychical mechanism of hysterical phenomena *(1893), Freud expanded on the concept of *traumatic neurosis*. The conceptual fluctuations of the word *trauma *in Freudian works suggest that the relative difficulties to establish this definition are much older than the conceptual confusion observed in PTSD. In his early papers, Freud used *trauma *as a key to explain the etiology of neurosis. From 1897 on, the concept loses importance as the concept of fantasy develops and takes the place previously held by traumatic events. However, the infamy of the First World War brought back the problems of "traumatic neurosis" to Freudian works, particularly in *Introduction to psychoanalysis and the war neurosis *(1919) [18] and in *Beyond the pleasure principle *(1920) [19].

*Trauma *was understood by psychoanalysis as an unspeakable experience, not elaborated, not signified, that was *incorporated *but could not be *introjected*, according to Nicolas Abraham (1919–1975) and Maria Torok (1925–1998) [20]. The barrier to symbolic elaboration is what assigns the traumatic quality to experiences. This approach reveals the point of view of psychoanalytical clinical practice: the expansion of the psychic creative and integrative dimensions may be the result of an elaborated, *introjected *trauma. In contrast, the paralyzing expansion of the *incorporated *trauma perpetuates the symptomatic reliving of suffering disconnected from language and, thus, far from the attribution of meaning to experience. Therefore, the way the event is treated or elaborated may become the traumatic element itself [21].

### Violence

*Violence *may be understood as the action of force or the act of violation. From Latin, *violentia *carries the broad meaning of behaviors with an origin in *vis *(force, vigor) and refers to "vehemence; passionate and uncontrolled force." Acts of excessive violence may result in the violation of rules, rights and norms, in which cases violence is understood as *violare *(violation, infraction) [22]. The definitions that combine the ideas of force and violation may increase the terminological confusion, because some acts of force do not result in the violation of norms, such as boxing fights, and some acts of violation do not require the use of force, such as the violation of human rights.

Violence may be initially understood according to a minimalist concept (violence as *violentia*), which is restricted to an act that should meet three conditions: deliberate attitude of the perpetrator, physical force, and destructive intent. *Episodic violence *corresponds to this concept and is characterized by the fact that it is direct and perpetrated fast and intermittently as an acute insult to a person's well being by means of a dramatic form of violence.

On the other side, the comprehensive concept (violence as *violentia*) includes psychic and subjective elements and stresses the victim's perspective. Its standard form is *structural violence*, an indirect form of violence whose norms are established socially and that is defined as a chronic insult to well being that kills or harms people slowly by continuous deprivation of basic human needs [22,23].

An example of a comprehensive concept of violence is found in the definition by the World Health Organization (WHO): "Violence is the intentional use of physical force or power, threatened or real, against oneself, another person, or against a group or community, that either results in or has a high likelihood of resulting in injury, death, psychological harm, maldevelopment, or deprivation" [24]. According to the WHO topology, violence may be self-directed, interpersonal or collective, and perpetrated by means of physical, sexual or psychological attacks, deprivation or neglect.

The WHO definition validates the concept of violence as an international, and not only local, problem, and prescribes the protection of vulnerable populations. However, the incorporation of the notion of *intention *adds complexity to this concept because intention is not always identifiable in a violent act [25]. The restriction of this concept to its intentional-actional aspect reduces the chances of considering the merely psychological dimension of some acts of violence, and invalidates the understanding that there are aggressive attitudes that lack a fully violent character [26].

The depth and breadth of the WHO definition are adequate to that organization's purposes, which require an ecological model of violence centered on multiple levels. However, when the breadth of what is denoted in a term expands, its descriptive power is retracted [27]. A comprehensive definition expands the use of the term "violence" to situations that result from economic poverty, social alienation, or political repression.

The application of this comprehensive concept of violence to PTSD would result in very permissive boundaries for the definition of a phenomenon as violent. Traumatic situations that have social, political or economic origins are beyond the reach of psychiatric and psychological treatments. Moreover, evil-minded individuals could distort the use of this broader sense of the word to claim medical benefits and secondary gains based on this "happy combination of a vague descriptive content and a negative emotional connotation" [27].

The concept of violence as force (minimalist conception) was refuted by Hannah Arendt, who established the distinction between power, potency (vigor), force, authority and violence. In the common sense, these terms are usually misunderstood or mistaken because are comprehended as a whole from the aspects of the domination of someone or something over others [28-30]. Arendt defines violence based on its merely instrumental property (depersonalization of violence), on the refusal of organicistic metaphors of violence (denaturalization of violence), and through the loss of the magical or demoniac characteristic that are commonly attributed to it (demythification of violence).

Arendt's theory of violence, although developed in the field of political science, also enriches the clinical care to victims of violence. The idea of denaturalization of violence destroys positivist references and inspires practices that go farther then a merely organicistic or psychologizing understanding of traumatic phenomena.

The notion of depersonalization of violence adds new meanings to the psychotherapy of domestic violence victims, who usually have ambiguous feelings for the perpetrators, which complicates their psychotherapeutic implication in the process of elaborating trauma. To understand violence by means of its instrumental character and not by personification of evil allows the victims to elaborate on their suffering based on the functions that violence operates in that relationship, and not based on a moral judgment of the aggressor.

Finally, demythification of violence indicates the "banalization of evil" and has clear implications on psychotherapeutic care because a barbarian type of violence is a barrier to the process of understanding the traumatic experience, as will be seen next.

### Barbarism

The first appearance of the word "barbarism", associated with rude, brutal and unintelligible speech, is found in Homer's *Iliad*. This term was used by ancient Greeks to refer to foreign peoples. The term, originally not disqualifying, referred to those that did not understand Greek and pronounced inarticulate and incomprehensible sounds, such as onomatopoeias: "bar-bar-bar". Barbarians only became dangerous and culturally inferior enemies after the Greco-Persian Wars (5th Century B.C.). The notion of barbarism as a clear opposition to civilization is assigned to Romans, who borrowed the term "barbarism" from the Greek and for the first time raised an insurmountable barrier between *Romans *and *Barbarians *[31].

Romans started using the term not only to describe peoples beyond their borders, but also for those in their own world who did not belong to the Greek-Roman cultural world [31]. Therefore, the meanings of barbarism were built in opposition to different understandings of civilization, in the sense of (a) civility; (b) historical and cultural background; or (c) humanity in a moral sense. Therefore, Barbarians were those who lived, respectively, in ancient, lower stages of (a) *socialization; *(b) *culture*; or, more importantly, (c) in a *pre-human *(savage) stage in relation to those that called them barbarians [32].

It is no longer startling that a highly refined and educated civilization may reach the worst of barbarism, such as in Nazi Germany, which used the advancement of their techniques and knowledge to exterminate human beings rationally and in an industrial scale. Therefore, the simple and single definitions of barbarism and civilization as opposites do not exist [32]. The term barbarism expresses agreement with the idea of civilization and, therefore, of superior and inferior cultures. Conversely, to accept a relativist position and to deny the concept of barbarism also poses difficulties. This point of view renders individuals enclosed in the specificity of their culture, and, therefore, the existence of universal values is denied. Therefore, those who spouse a relativist position would not be able to fight "barbarian" practices because they understand that cultures are equal in their right to express their habits and customs [32].

This dialectical trap may be avoided by defining a culture as barbarian if it lacks structures to recognize the alterity of things, by defining customs as barbarian if their effects deny a specific form of human existence, and by describing individuals as barbarian if they are incapable of tolerating diversity. The meaning of barbarism is more clearly defined when a culture is analyzed in relation to itself, not by classifying as barbarian those who were left out of any civilization process, but by recognizing barbarism when people fall behind, in a peculiarly hideous way, their own civilization even though this civilization has achieved the highest levels of development [33].

In general, terrorist attacks can be considered as one specific form of barbarism, once "attackers tend to use horrific violence to cause massive destruction and death and to use other tactics (e.g., biological weapons) to terrify the public" [1], and mostly innocent civilians in nonwar zones. Recent surveys on the impact of international terrorism on mental health points to the important aspect that different forms of violence tend to induce different impacts on mental health [1].

The terrorism and other barbarian aspects of violence imply a lack of meaning rather than some symbolic construction that "justifies" the traumatic event that affected the victims or their relatives. Finding a "justification" for violence outlines a symbolic shield against terror, the initial mechanism of assigning meaning to traumatic experiences. Conversely, lack of meaning is the trademark of barbarian violence and an obstacle to the elaboration of trauma and its consequent symbolic integration in the victim's life. This significant gap is filled by the abundance of symptoms that result from the persistence of the traumatic memory.

### Conceptual developments

The tenth edition of the International Classification of Diseases (ICD-10) and the fourth edition of the Diagnostic and Statistical Manual of Mental Disorders (DSM-IV) describe similar criteria to diagnose PTSD. According to criterion A of DSM-IV, a diagnosis of PTSD should be made when "the person has experienced, witnessed, or been confronted with an event or events that involve actual or threatened death or serious injury, or a threat to the physical integrity of oneself or others" (criterion A1), and whose response to the event "involved intense fear, helplessness or horror" (criterion A2) [34]. According to the ICD-10 guidelines, "the patient has been exposed to a stressful event or situation (either short- or long-lasting) of an exceptionally threatening or catastrophic nature, which is likely to cause pervasive distress in almost anyone" [35].

The historical trajectory of the concept of PTSD, from DSM-III to the revised edition of DSM-IV, attests to "the centrality of the stressor criterion in the definition of this disorder" [36]. In the DSM-IV, there is an attempt to objectively define the "traumatic event", as it clearly establishes that the stressor is limited to experienced or witnessed situations that necessarily involve *death*, serious *injury *or a threat to *physical integrity*. In regard to the first stage of the process of stress – the isolated environmental input – the criterion A1 indicates a return to the older meaning of the word "trauma", which refers exclusively to a *bodily wound*, as originally used in medicine.

The second stage in the process of stress can be found in criterion A2 of the DSM-IV, and reflects the psychological dimension of trauma through the immediate apprehension of the event by the victim in the form a response of "intense fear, helplessness or horror." Some authors question the restriction of criterion A2 to only these trauma-related emotions, "when there is recent evidence that both anger with others and shame" are also "strong predictors of PTSD symptoms longitudinally" [37].

The criterion A1 excludes the situations that do not involve direct physical violence or "threat to physical integrity", but that are capable of producing psychopathological responses of reliving, avoidance, and hyperarousal. The point here is not to artificially expand the criterion to include the more questionable dimensions of a broader concept of violence. The purpose is to rectify, from a nosographic perspective, the conclusion that, if an event can be simply characterized as a "threat", such threat may be defined not only as physical threat, but also as moral coercion, psychic intimidation, or symbolic coercion. A good example of such is the development of PTSD by some victims of bullying, a type of violence that is perpetrated not necessarily and only by means of threat to physical integrity, but that may occur by means of systematic psychological attacks that take the form of verbal offense, acts with the intent to ridicule in front of others, and social isolation from youth groups with certain physical or psychic characteristics.

Furthermore, in the context of ongoing terrorist threats, especially after the 9/11 attacks, the presence of widespread posttraumatic stress disorder symptoms among individuals not directly exposed to the attacks was documented and replicated in independent cross-sectional and longitudinal studies [38]. This *indirect exposure*, largely through the media, questions the "perhaps outdated stimulus-response model" based on the "conventional assumptions that a disaster should be defined as local and must be directed experienced to cause psychopathology" [38].

The fragility of the ICD-10 approach to the event itself is shown, initially, in the circularity of its definition, once it is based on the response elicited, which leads to a tautology: to develop posttraumatic *stress *disorder, the patient should have "been exposed to a *stressful *event or situation." Besides, the description of the nature of this *stressor *as "something *exceptionally *threatening or catastrophic" evokes the conceptual weakness of this criterion rather than is an attempt to outline or characterize the type of event. After all, if the notion of *exceptionality *refers to something that is "out of the ordinary" and that "occurs *beyond the limits of what is usual*, normal, frequent or ordinary" [9], what would these limits be, and by whom would they be established?

Moreover, this construct becomes even more complex when analyzed according to an individual perspective. A person with PTSD symptoms may have perceived as *exceptionally threatening *a situation that might not cause pervasive distress "in almost anyone." The perspective adopted in the ICD-10 is in disagreement with a decisive factor in clinical practice: for patients that present typical PTSD symptoms, the traumatic factor is not necessarily identified in the reality that they can share, but maybe only in their personal and subjective experiences.

The conceptual inaccuracy of criterion A of the ICD-10 may also be found in the specification of the nature of the event as "catastrophic" or "threatening". How to discuss, then, the barbarian forms of violence that are not a catastrophe or do not constitute a direct threat, but that are capable of triggering PTSD? Barbarism may be described as a type of violence that, in some cases, prescinds from the association with "exceptional threat" for its perpetration. Barbarism may simply be a paroxysmal form of violence, an act without warning, without threat, whose only objective is destruction and death of another human being.

Not infrequent are cases of people that get together to perpetrate acts of violence against homeless, black or native people, gays or members of other minorities by ambushing the victims, sometimes when asleep, without any type of previous threat. Because of the barbarian death of one of their members, these minorities may develop chronic hyperarousal, avoidance and intrusive thoughts of situations associated with that barbarism, and, therefore, meet all the criteria for a PTSD diagnosis although they have not concretely experienced a direct threat. In such situations, something that should be perceived as normal by most anyone, such as belonging to a minority group (blacks, natives, gays), is experienced as an "exceptionally threatening" situation.

In the same manner, "there are now replicated findings that PTSD symptoms related to the September 11, 2001, attacks occurred in large numbers of persons who did not fit the traditional definition of exposure to a traumatic event" [38]. It is like if individuals were all the time perceiving this "exceptionally threatening" overall. Therefore, in face of such considerations, how can a limit be established between what would or would not cause this "exceptionally threatening" or this "pervasive distress in almost anyone"?

A careful analysis of the criteria currently used by the APA and WHO psychiatric guidelines shows inaccurate definitions of violence, disaster, catastrophe, trauma and barbarism. Such critical appraisal should inspire new studies to improve diagnostic and therapeutic parameters used in clinical practice.

In the analysis of the DSM-IV criteria, such improvement should focus on the fact that this classification limits the real event or threat to the *physical aspect *of trauma. According to the considerations above, violence prescinds from force and physical damage, and the psychological dimension of a threat may be the most devastating face of violence. The restriction of this criterion to the minimalist concept of violence may exclude diagnoses and treatment of a significant number of patients with PTSD. At the same time, a broad understanding of violence exceeds the limits of clinical practice, as demonstrated in the discussion of the concept established by the WHO. Between these two concepts, there is an alternative: to include the aspect of psychological violence as one more possible trigger of PTSD without expanding the concept to the point of introducing aspects that are too broad for clinical practice.

The ICD-10 also shows misconceptions in its criterion A, such as the generalizing definitions discussed above. New definitions of this diagnostic category should necessarily include the "impossibility of symbolic elaboration" as a condition for the development of this disorder. This is a dimension that includes the senses of "trauma", "barbarism" and, many times, "catastrophe", and replaces vague expressions such as "exceptionally threatening."

The difficulty in understanding and elaborating an event adds an important diagnostic value to this criterion because it assigns priority to a person's individual psychic response rather than to the effects found in "almost anyone." This latter expression is not only imprecise, but may also exclude PTSD cases that, although originated in individual situations, may result in psychic sequelae of traumatic characteristics.

In summary, the aspects discussed may be organized as a blueprint for the changes in criterion A of the diagnostic classifications as follows:

A) Delayed psychic response to a situation or event (either short- or long-lasting) whose understanding or symbolic elaboration is not possible for the person because of the magnitude of physical or psychological threat or the actual presence of death, injury or severe psychological distress, with an immediate response of intense fear, helplessness, dissociation or horror.

### Final considerations

The examination of PTSD terminology defined two completely different tasks: the understanding of the meaning of the expressions used by the patient; and the search for terms that translate such expressions into medical language. The "disaster" described by the patient may be only an unexpected experience that requires an intense existential implication for which this person does not feel prepared. A situation referred to as "traumatic" raises the examiner's interest in obtaining details of the circumstances of the event described because the extension and intensity of the stimulus may reflect differently in psyche. A more severe psychopathological presentation is marked by silence and difficulty in elaboration, which may indicate experiences of severe contact with a form of violence, such as barbarism.

The purpose of clarifying the terms used in the field of PTSD is to provide a resource for conceptual clarity, terminological precision, and understanding of meaningful nuances of the words used in clinical practice. Patients seen after an experience of imminent death do not need therapists that operate according to a good-versus-evil system, or who assign all evil to the perpetrator and reinforce the patient's role as a victim. These patients have already "faced death" and, therefore, therapy should not be conducted through the personalization of violence; it is not the positivist naturalization of violence that provides the theoretical basis for the clinical treatment of PTSD; and, finally, it is not the mythification of violence that will enable society to overcome this phenomenon.

## Summary

1. The plethora of terms used in PTSD literature does not reflect the understanding of meanings, applications and limits of these concepts.

2. The "disaster taxonomy" indicates event characteristics that may generate different psychic responses and, therefore, requires precise terms to describe the three stages of the process of stress.

3. Because of changes in the concept of trauma along time, it can now be understood in its integrative or paralyzing psychic dimensions depending on how the traumatic experience is elaborated.

4. The use of a broad concept of violence in PTSD leads to questions out of the range of psychotherapy, and the barbarian type of violence functions as a barrier to resilience.

5. The criterion A of the ICD-10 and DSM-IV show conceptual imprecision and fragilities that may have effects on the diagnosis and treatment of victims of violence.

## Competing interests

The authors declare that they have no competing interests.

## Authors' contributions

LLB conceived of the study, performed the literature search, participated in the interpretation and discussion of data, drafted and wrote the manuscript. JPF performed the literature search, participated in the interpretation and discussion of data, coordinated the multiple revisions of the manuscript. MFM conceived of the study, participated in the interpretation of data and coordinated the multiple revisions of the manuscript. JJM participated in the interpretation of data and critically revised the first versions of the manuscript. All authors read and approved the final version of the manuscript.

## Pre-publication history

The pre-publication history for this paper can be accessed here:


